# Rheumatoid arthritis in Latin America: the importance of an early diagnosis

**DOI:** 10.1007/s10067-015-3015-x

**Published:** 2015-07-25

**Authors:** Licia Maria Henrique da Mota, Claiton Viegas Brenol, Penelope Palominos, Geraldo da Rocha Castelar Pinheiro

**Affiliations:** Serviço de Reumatologia, Hospital Universitário de Brasília, Universidade de Brasília (UnB), Campus Universitário Darcy Ribeiro, Brasília, Distrito Federal 70910900 Brazil; Hospital de Clinicas de Porto Alegre, Universidade Federal do Rio Grande do Sul (UFRGS), Ramiro Barcelos 2350, Porto Alegre, Rio Grande do Sul CEP 90035-903 Brazil; Rheumatologist, Rheumatology Division, Hospital de Clinicas de Porto Alegre, Federal University of Rio Grande do Sul (UFRGS), Ramiro Barcelos 2350, Porto Alegre, CEP 90035-903 Rio Grande do Sul Brazil; Universidade Estadual do Rio de Janeiro, Boulevard 28 de Setembro, 77 sala 333 - CEP 20551-030 Rio de Janeiro, Rio de Janeiro Brazil

**Keywords:** Early, Difficulties, Inequality, Latin America, Rheumatoid arthritis, Solutions

## Abstract

The generalization of the early rheumatoid arthritis (ERA) concept and the existence of a window of therapeutic opportunity—a time span in which the institution of a proper therapeutic method for the disease would determine clinical improvement—have set the notion that early diagnosis and treatment may modify the course of the disease. Although in several regions of the world, especially in North America and Europe, since the year 2000, a significant reduction in diagnostic delay was observed in cohorts of patients with rheumatoid arthritis (RA), probably reflecting a stronger awareness of the importance of early diagnosis, this is not a reality in Latin America (LA). LA is a region of great economic inequality, with disparities in access to the public healthcare system and limited access to private medicine, being widely difficult to obtain a specialized medical evaluation in both scenarios. This paper aims to briefly review the main difficulties in the management of ERA in LA, based on the review of the literature, on the evaluation of a survey conducted among 214 rheumatologists of LA, members of Pan-American League of Associations for Rheumatology (PANLAR) and the experience of the authors. The paper also aims to propose solutions to the difficulties in managing ERA in LA.

## Introduction

Rheumatoid arthritis (RA) is an autoimmune, chronic inflammatory disease characterized by joint swelling, joint tenderness, and destruction of the synovial joints that can lead to severe disability and premature mortality. RA affects between 0.5 and 1 % of the general population, mainly during their working age, affecting, thus, the functional capacity, with great economic burden to the individual and the society [1].

In the last decades, there was a clear evolution in knowledge about physiopathology of the disease, resulting in changes in its approach and treatment. The association between symptom duration and RA persistence is not linear, suggesting the presence of a confined period in which RA is more susceptible to treatment [2].

Early RA (ERA) is defined as the diagnosis given in the first weeks or months of joint symptoms or signs. The generalization of the ERA concept and the existence of a window of therapeutic opportunity—a time span in which the institution of a proper therapeutic method for the disease would determine clinical improvement—have set the notion that early diagnosis and treatment may modify the course of the disease [3].

Currently, evaluating a patient with articular symptoms in the first possible opportunity is aimed at, and the definition of the RA’s early stage comprises the first weeks or months of symptoms (generally less than 12 months), the first 12 weeks of manifestations with very early rheumatoid arthritis (VERA) standing out as a critical period. Those patients with more than 12 weeks and less than 12 months of articular symptoms are included in what is known as late early rheumatoid arthritis (LERA) [4].

Concomitantly, laboratory and imaging methods were improved or developed, contributing to an earlier diagnosis and determination of prognostic for early RA [5]. In the last two decades, RA’s treatment has undergone intense changes, reflecting both the modification of the therapeutic approach paradigm and the introduction of new classes of disease-modifying anti-rheumatic drugs (DMARDs), including biological response modifiers (biological therapy) [6].

In this context, early arthritis recognition clinics (EARC) and ERA clinics have been established in some countries in recent decades, in order to receive early and offer appropriate treatment for patients within the first months of disease evolution, including medical follow-up by the rheumatologist and, ideally, multidisciplinary care [7].

Despite these progresses, it is well known that the limiting factor to a good therapeutic response is still the delay in diagnosis and in the institution of the adequate treatment, as well as the difficulty in handling the medication during the patient’s follow-up [8].

Although in several regions of the world, especially in North America and Europe, since the year 2000, a significant reduction in diagnostic delay was observed in cohorts of patients with RA, probably reflecting a stronger awareness of the importance of early diagnosis, this is not a reality in Latin America (LA) [9–12].

LA and the Caribbean, a rapidly growing region with almost 600 million inhabitants composed of Mexico, Central and South America, and the islands of the Caribbean, is one of the most unequal regions in the world, according to the Economic Commission for Latin America and the Caribbean (United Nations) [13, 14]. People from different LA countries have little access to private medicine and disparities in access to the public healthcare system, with widespread difficulty to obtain specialized medical attention in both.

This paper aims to briefly review the main difficulties in the management of ERA in LA, based on the review of the literature, the evaluation of a survey conducted among Latin American rheumatologists members of Pan-American League of Associations for Rheumatology ) and the experience of the authors. The paper also aims to propose solutions to the difficulties in managing ERA in LA.

## Materials and methods

We searched PubMed, Lilacs (Latin American and Caribbean Literature on Health Sciences), and Scielo (Scientific Electronic Library Online) using terms related to early arthritis and, in PubMed, combining them with country names or regional names related to Latin American and the Caribbean Islands. The search strategy for PubMed was: (early arthritis OR recent onset arthritis) AND (“Latin America” OR “Central America” OR “South America” OR “Caribbean” OR “Argentina” OR “Bolivia” OR “Brazil” OR “Chile” OR “Colombia” OR “Costa Rica” OR “Cuba” OR “Dominican Republic” OR “Ecuador” OR “El Salvador” OR “Guatemala” OR “Haiti” OR “Honduras” OR “Mexico” OR “Nicaragua” OR “Panama” OR “Paraguai” OR “Peru” OR “Uruguay” OR “Venezuela”). In Lilacs, we preceded two searches, using the terms “early arthritis” and “early rheumatoid arthritis.” To search through Scielo, we used the terms “early arthritis” (without quotes) and “recent onset arthritis.” Articles were selected when they approached early arthritis clinic or management of early arthritis in Latin America and the Caribbean.

Aiming to know the panorama of the difficulties relating to the management of ERA in LA, a survey was conducted among members of PANLAR. The questionnaire was forwarded by e-mail to all members of PANLAR, containing, among other evaluations, 20 specific questions about ERA. Several themes were addressed, including the existence or not of ERA clinics or EARC in the region (and the country), the definition of ERA (duration of symptoms), the system of reference from basic assistance, and the main difficulties for access of patients to diagnostic and therapeutic management. It was also requested from rheumatologists possible solutions to the problems raised.

Some of the questions were multiple choice, while others demanded detailed answers. The questionnaire is available as Appendix 1.

## Results

A total of 438 articles were identified through the online search: 304 in PubMed, 33 in Lilacs, and 106 in Scielo. Some of them were excluded as duplicates and most were excluded after screening title. Around 50 articles were selected to full-text reading and 13 were used as references for this paper.

Two hundred and fourteen rheumatologists, from different LA countries, answered the survey. Table 1 summarizes the responses of Latin American rheumatologists to multiple-choice questions. Table 2 present aspects of the current reality found on the Latin American countries, listing the challenges and proposed solutions for resolving the difficulties observed, based on the interpretation of the questionnaire applied (detailed answers) regarding the importance of the early diagnosis of RA in this region.

**Table 1:** Summary of the responses of rheumatologists to multiple-choice questions to know the panorama of the difficulties relating to the management of ERA in LA

Early rheumatoid arthritis scenario in Latino America		
Questions	Answers	*N* (%)
In there any ERA or early arthritis clinic in your practice?	Yes—there is ERA clinic	27 (13.24)
	Yes—there is early arthritis clinic	41 (20.10)
	No	136 (66.7)
If the answer to the first question is negative, where ERA or early arthritis patients are evaluated?	In established RA clinic	37 (52.9)
	Other	53 (38.41)
If the answer to the first question is positive, how long does it exist?	<1 year	10 (13.7)
	Between 1 to 3 years	22 (13.14)
	Between 3 to 5 years	17 (23.29)
	Between 5 to 10 years	20 (27.4)
	More than 10 years	4 (5.48)
To be considered ERA, how long symptoms need to be present?	Less than 12 weeks	81 (41.75)
	Less than 1 year	90 (46.39)
	Less than 2 years	23 (11.86)
	Less than 3 years	0 (0)
In your practice, do you use any classification criteria for diagnosis of ERA?	No	22 (11.06)
	Yes, American College of Rheumatology (ACR) 1987 criteria	8 (4.02)
	Yes, ACR/EULAR 2000 criteria	166 (83.42)
	Other	3 (1.51)
In your outpatient care, how many patients are you following with ERA?	Less than 50 patients	80 (48.78)
	Between 50 and 100 patients	55 (33.54)
	Between 100 and 200 patients	17 (10.37)
	More than 200 patients	12 (7.32)
How many professionals work in the ERA clinic?	Professors	32 (17.58)
	Rheumatologists	171 (93.96)
	Residents	77 (42.31)
	Physicians (not rheumatologists)	20 (10.99)
	Nurses	38 (20.88)
	Occupational therapists	31 (17.03)
	Administrative staff	25 (13.74)
	Other	30 (16.48)
How is the access of the patients to ERA clinic?	Free access	95 (48.47)
	Referral by general practitioners	122 (62.24)
	Referral by rheumatologists	29 (14.80)
	More than one above	52 (26.53)
	Other	8 (4.08)
Is there any endemic disease in your country important for differential diagnosis of RA?	Yes	31 (15.35)
	No	171 (84.65)
What composite score do you use in your practice?	DAS28	192 (94.58)
	HAQ	116 (57.14)
	SDAI	27 (13.30)
	CDAI	54 (26.60)
	None	5 (2.46)
	Other	12 (5.91)
-What questionnaire of quality of life do you use in your practice?	HAQ	179 (90.40)
	MHAQ	13 (6.57)
	RAPID	25 (12.63)
	Other	8 (5.05)
What kind of treatment patients are using in your practice?	Glucocorticoids	105 (51)
	Non steroidal anti-inflammatories	125 (61)
	Synthetic DMARDs	179 (87)
	Biologic DMARDs	20 (10)
How often do patients with ERA visit the physician?	Once in a month	57 (29.23)
	Every 3 months	99 (50.77)
	Every 6 months	12 (6.15)
	Once in a year	4 (2.05)
	Other	23 (11.79)

**Table 2:** The importance of early diagnosis of rheumatoid arthritis—current reality, challenges, and proposed solutions

Current reality	Challenges	Proposed solutions
- There are few structured clinics for screening (capitation?) and follow-up of patients in the early phase of arthritis or RA	Creation/establishment of clinics with appropriate structure to receive early on, diagnose and treat early arthritis, both in public or private services	- Establishment of guidelines (and guides) for the implementation of early arthritis clinics in each service (by the local rheumatology associations basing themselves on successful experiences on the region itself) - Awareness of the population, health managers, and government on the importance of the early diagnosis and the existence of specific structures for diagnosis and follow-up
- Lack of medical professionals (rheumatologists) on the existing clinics	- Allocate rheumatologist to outpatient early arthritis or early rheumatoid arthritis clinics	- Establish healthcare policies, on public and private services, that recognize the importance of allocating rheumatologists in reference units to diagnose and follow-up on early arthritis; - Continuing medical education ofrheumatologists and engagement in early RA clinics
- Shortage of other healthcare professionals (nurses, occupational therapists, physiotherapists, physical educators, psychologists, social workers, nutritionist) and supporting staff (secretaries) at the existing clinics	- Designate healthcare professionals of correlate areas for the care of patients with early arthritis or early RA	- Establishment, on RA patients’ follow-up protocols, of the importance of multidisciplinary follow-up - Engaging of other professionals for patient follow-up
- Patients’ difficulty of access to arthritis clinics or early arthritis clinics, both on public and private services - Patient lateness in seeking health assistance *Geographical factors (great distances—patients that reside in rural areas, far away from centers where there are rheumatologists) *Cultural factors (fear of the diagnosis, belief in a “serious and incurable disease”) *Social and economical factors (lack of resources for transportation or access to the doctor on the private system) *Self-medication (frequent use, with free access, and no prescription, of anti-inflammatory drugs and corticosteroids) *Delay on the referral from other specialists (general practitioners, orthopedists) *Inexistence of an adequate reference/counter reference system *Lack of vacancies for the appointment of new cases	- Optimize patients’ access to reference centers for diagnosis and treatment of early RA - Reducing the time between the onset of symptoms and the assessment by the rheumatologist - Improve the level of knowledge on RA of the general practitioner and of other correlated specialist	- Educational campaigns for the patient (information on the alert symptoms of RA and on the importance of the early diagnosis/treatment) - Education and training of general practitioners and orthopedists (adequate training of general practitioners, general internists, and allied health professionals in articular examination and RA symptoms and the creation of EAC or areas within rheumatology units to provide care for patients referred early in the course of their disease) - Inclusion of information on arthritis on the medical course (graduation) - Establishment of a efficient system of reference/counter reference that allows the referral of a patient by the general practitioners or orthopedist in a timely fashion, with a posterior referral to the primary health care unit, after controlling the disease
-- Difficulties to diagnose RA -- Restricted general access to additional tests (such as inflammatory activity tests, rheumatoid factor and ACPA) and some image exams (ultrasonography and magnetic resonance imaging) on public services, and high cost on private services -- Lack of diagnosis protocols	- Broaden the access to additional tests eventually necessary for the differential diagnosis, both on public and private healthcare services	- Establishment of local protocols (regional associations of rheumatology, hospital services protocols) for early diagnosis of RA - Inclusion of additional tests in the public services paying lists and the private paying sources
- Difficulties for the differential diagnosis: - Occurrence of other infectious conditions that have differential diagnosis with RA (Chikungunya fever, Hansen’s disease, dengue fever, CMV, parvovirus B19, HIV, viral hepatitis, tuberculosis) - Frequently limited access to viral serology and other tests for differential diagnosis	- Broaden the access to additional tests and image exams eventually necessary for the differential diagnosis, both on public and private healthcare services	- Inclusion of possible endemic-epidemic diseases on Latin America as possible differential diagnosis for the initial arthritis cases - Inclusion of the necessary additional tests for differential diagnosis in the public services paying lists and the private paying sources
- Non application or non existence of protocols for treating early RA - Difficulty of access to synthetic and biological medication	- Dissemination and application of the existing protocols, or creation of regional protocols for treating early RA - Extend the access to synthetic medication and, when necessary, biological	- Many of the existing protocols and guidelines, including the local Latin American associations’ protocols, already contemplate treatment for early RA, being necessary a greater dissemination of those among rheumatologists and correlated specialists. - Inclusion of DMARD in the public services paying lists and the private paying sources, with emphasis on the importance of early therapy
- Difficulty to maintain an adequate periodicity between appointments due to lack of professionals and vacancies	- Implementation of circumstances that enable the creation of new vacancies on existing early arthritis clinics	- Establishment of an effective counter reference system for discharge of patients followed at early RA clinics

## Discussion

According to Community Oriented Program for Control of Rheumatic Diseases (COPCORD) methodology, musculoskeletal (MSK) complaints in the last 7 days, unrelated to trauma, are common but vary among different populations [15]. In LA countries, the prevalence of MSK complaints was as low as 9.3 % in Guatemala [15–17], 25.5 to 27.1 % in Mexico [15, 18–20], 26.9 to 30.9 % in Brazil [15, 21, 22], and as high as 43.9 % in Cuba [15, 23], 45.1 % in Chile [15], and 46.5 % in Peru [15]. In most studies, a higher prevalence of pain unrelated to trauma was detected among females [15].

Ideally, patients with recent onset of MSK pain should be seen by a primary care physician (PCP) (general practitioner—GP). On the other hand, in the presence of signs or symptoms of systemic or inflammatory disease, these patients should promptly be referred to a rheumatologist. The proportion of rheumatologists that have the opportunity of evaluating a patient in the first 6 weeks of symptoms has gone from 9 % in 1997 to 17 % in 2003, although not all of the cases may be evaluated so quickly [24].

Unfortunately, the early referral to a rheumatologist is not what usually happens in most, if not all LA countries [25, 26]. This might be due to several reasons:

1. Most people do not realize that “rheumatism” is a vague term that encompasses many diseases with different treatment and prognosis, so they are not aware that the first thing they need to do is to have a diagnosis to explain their MSK condition;
2. Many people do not have easy access to the healthcare system, delaying the medical evaluation;
3. Most patients with MSK pain are referred by the GP to the specialist without a specific referral criteria (most patients with common diseases such as osteoarthritis, fibromyalgia, and mechanical low back pain should not be referred to a rheumatologist but followed by a GP);
4. Many GP do not readily identify the patients with inflammatory conditions delaying the proper referral to the specialist;
5. There are few rheumatologists in the secondary or tertiary healthcare system so it usually takes some time for this specialist’s appointment;
6. Most, if not all, rheumatology outpatients clinics do not have a triage system or a “fast track agenda” for early arthritis patients (early arthritis clinic—EAC) so even those patients need to wait to be seen.

Early recognition and treatment with DMARDs is important in achieving control of disease and prevention of joint injury and disability—this strategy is associated with improved clinical and radiographic outcome. The first 12 weeks of symptoms, in particular, are a critical period called as “very early RA” (VERA) and represents the best chance to achieve a complete remission and to stop the erosive course of RA [27–29]. Not all patients meet the criteria for RA at the early stages of the disease. In clinical practice, all cases of arthritis that cannot be classified in one of the accepted categories are referred as “undifferentiated arthritis” (UA). In one third of patients with recent onset arthritis, it is not possible to come to a definitive diagnosis at presentation. Among these cases, approximately 30 % will progress to RA [30]. The others may have alternative definitive diagnoses such as infections, spondyloarthritis, other systemic rheumatic diseases, microcrystalline arthropathies, osteoarthritis, or others; may also evolve into remission or even remain as UA [30, 31].

There seems to be, however, a consensus among LA key opinion leaders rheumatologists about the importance of early diagnosis for the proper management of RA by rheumatologists [24–27]. There is evidence of systematic differences between rheumatologists and non-rheumatologists in early recognition and in initiating the use of DMARDs for treatment of RA. Non-rheumatologists generally delay treatment, resulting in worse outcomes of the disease [32] RA patients were diagnosed earlier, receive DMARD therapy more frequently and achieved better clinical and radiographic outcomes when managed by rheumatologists [32–35]. To the best clinical and functional prognosis of patients with RA, it is recommended that the primary care physician refer briefly suspected cases to the rheumatologist [36]. Despite this, the average time for the first visit of RA patients with a rheumatologist is 17 and 19 months to elapse before the first administration of DMARDs [37].

The diagnosis of RA is established considering clinical findings and complementary examinations. No isolated test, laboratory, imaging or histopathological, confirms the diagnosis alone.

There have also been “proposed actions” to increase the early suspicion, the proper diagnoses of RA, and right and rapid referral of these patients to the rheumatologists [38]. Among other ideas, it was proposed an adequate training of general practitioners, general internists, and allied health professionals in articular examination and RA symptoms and the creation of EAC or areas within rheumatology units to provide care for patients referred early in the course of their disease.

The gap between recognition of symptoms, diagnosis, and treatment is dependent at least from four steps: from the patient at symptom onset to assessment in primary care, from primary care provider (PCP) to rheumatology referral, from rheumatology referral to assessment and from rheumatology assessment to commencement of DMARD therapy. Several strategies have been studied with the aim of reducing the interval between each of these four steps, including the training of primary care physicians for the early recognition and referral of suspected cases of RA, self-administered questionnaires, triage of referrals, triage clinics and early arthritis clinics (EACs) [39].

Only few studies carried out with Latin American populations on the demographic and clinical characteristics of patients diagnosed with ERA can be found in the literature.

GLADAR (*Grupo Latino Americano de Estudio de Artritis Reumatoide*) was a large, multicenter, multinational inception cohort of Latin American patients. Consecutive patients with ERA (<1 year of disease duration as diagnosed by a rheumatologist) from 46 centers in 14 Latin American countries were enrolled in GLADAR. Clinical data, laboratory assessments, and a detailed registry on type of prescription of 1093 patients were collected at baseline and at 3, 6, 12, 18, and 24 months of follow-up. GLADAR has issued some guidelines for the pharmacological treatment of RA that promote an early aggressive therapy. Early disease clinics are established in some LAC countries. The GLADAR experience has shown that most early RA patients (i.e., <1 year of disease duration) receive methotrexate [40–42].

CONAART (Consorcio Argentino de Artritis Temprana—Argentine Consortium for Early Arthritis) is an initiative of seven rheumatology centers across Argentina. Patients were included if they had at least one or more swollen joints and <2 years of disease duration. A total of 413 patients were included. From CONAART data, we know some social, demographic, familiar, hereditary, clinical, and laboratory data from Argentine ERA patients [43]. CONAART also informed about work disability and its main associated factors in patients with ERA [43]. They also analyzed the effects of cigarette smoking on disease activity, functional capacity, radiographic damage, serology, and presence of extra-articular manifestations in patients not only with ERA, but also with undifferentiated arthritis, and found that neither was tobacco exposure related to the presence of extra-articular manifestations or to the degree of disability in any of the two groups of patients [44].

The Brasilia Cohort of RA is an incident cohort of patients with early RA, followed in the Outpatient Rheumatology Clinic of Hospital Universitário de Brasília, Universidade de Brasília. For inclusion in this cohort, early RA is defined as the occurrence of joint symptoms compatible with pain and joint swelling with an inflammatory pattern, with or without morning stiffness or other manifestations suggestive of inflammatory joint disease, assessed by a single observer, lasting more than 6 weeks and less than 12 months, regardless of meeting the criteria of the American College of Rheumatology (ACR). All selected patients retrospectively met the EULAR/ACR criteria 2010. From the moment of diagnosis, patients are followed prospectively, and receive the standard treatment regimen used in Brazil, including synthetic or biological DMARDs, according to their needs. Patients are monitored in accordance with the principles of Treat to Target, and the medication adjusted to achieve remission. Currently, there are 132 patients accompanied by protocol form for up to 11 years, from the initial diagnosis. From Brasilia Cohort, we have information about demographic, clinical, laboratorial and radiographic characteristics of patients enrolled in this Brazilian cohort, disease activity, and impact on quality of life. Other aspects also have been studied by Brasilia Cohort, including, for example, physical activity practice among patients with early RA and the possible association between physical activity, disease activity and functional disability, frequency of vaccination, and the orientation about vaccines among patients in Brasilia Cohort [45–49].

Solutions are needed to offer patients with inflammation of the small joints— with a high likelihood of having rheumatoid arthritis—to be seen quickly and receive treatment in a timely fashion. For early recognition and management of RA, efforts must be composed mainly of medical education and health system organization. As a feasible example in LA, a network for early diagnosis and management of arthritis was implemented adopting several strategies: primary care providers education, use of referral algorithms, creation of a rapid access system to an early arthritis clinic (EAC) and institution of a task force to reduce the waiting list for rheumatologist assessment. Through an initiative involving Primary and Tertiary Public Health Care Centers in the South of Brazil, general practitioners were capacitated to recognize and provide early referral to patients with arthritis and an EAC was created to offer prompt evaluation of newly referred patients. The project was composed by two steps: medical training workshops for PCPs focused on early recognition and referral of patients with arthritis and the creation of an EAC for prompt treatment of newly referred patients. Several workshops for primary care physicians training were conducted in an University Hospital in South of Brazil, the Hospital de Clínicas de Porto Alegre (HCPA). Each workshop lasted 4 h and on each session, three rheumatologists from HCPA ministered theoretical lesions which included several themes: concept and etiology of early UA, importance of early referral to rheumatologist to optimize outcomes, consequences of RA on function and quality of life, the algorithm for early referral proposed by the European League against Rheumatic Diseases (EULAR) [36], the use of DMARDs, focusing on methotrexate use, and the prevention and management of comorbidities in patients with RA. Each workshop also included a practical lesson where physicians assessed disease activity in RA by using composite scores: the Disease Activity Score 28 joints (DAS 28) [50] and the Clinical Disease Activity Index (CDAI) [51]. Fifteen students from Medicine School/Federal University of Rio Grande do Sul (UFRGS) were trained and participated as monitors during the practical lessons. Physicians participating in workshops were invited to visit the RA clinic in HCPA to have contact with RA patients, learning to recognize the presence of arthritis on physical exam. Additionally, a Clinic for Assessment and Treatment of Early Arthritis was created in a Tertiary Health Center (HCPA) after negotiations with the Municipal Health Office. Since then, four clinical consultations are offered each week for patients with early arthritis referred from PCPs. Patients referred to this clinic are seen in a timely fashion (2–4 weeks) because they are not sent to the usual referral system. The project allowed better recognition of patients with early arthritis and decreased referrals of patients who could be managed in primary care. The work is ongoing and was partially funded by PANLAR.

In Mexico, Sánchez et al. described the prevalence of dyslipidemia, serum lipid behaviour, and predictors of serum lipid levels in a cohort of 146 Mexican mestizo ERA patients [52].

Although there are not many publications on early RA clinics in Latin America, we do know that there are rheumatology services that maintain clinics with this purpose. On a survey performed among Latin American rheumatologists, with an Internet questionnaire sent by PANLAR, from 214 rheumatologists who responded this survey, 13.2 and 20.1 %, respectively, informed that ERA clinics or EARC do exist in their services.

A common pattern for social policies was developed in most Latin American countries, rooted in a similar development model and responsible for some of the most remarkable features of the relationship between the state and the society, as well as for incorporating a particular power structure into a formalized system. The general characteristics of this pattern were identified in the health sector as: the segmentation and/or exclusion of groups of the population; the fragmentation of the institutions; the narrow and fragile financial basis of the system, mainly based on contributions upon salaries, and the existence of strong actors with vested interests represented in this political arena. In spite of these similarities, it is indispensable to highlight the variations among the countries, concerning the way they faced this critical period and the effects of the adjustment policies on their recovery [53]. Generally, the public health system in LA countries serves the entire population. In the usual referral system, general practitioners can refer patients to specialists in a hospital setting or specialized outpatient clinic. Since there are few rheumatologists in the public system, patients have to wait their turn for the availability of a rheumatology consultation. This process takes months and, sometimes, even years. For patients with RA, this delay may represent the loss of the window of opportunity for therapeutic intervention.

On LA, just as in other regions of the world, the costs related with RA are elevated. Spending on RA patients assume greater impact in developing countries, where financial resources for health are less robust. This emphasizes the importance of studies assessing the costs and the allocation of resources. The diagnosis of RA in the initial phase is extremely important because appropriate treatment early in the disease can change the natural history of this condition.

The answers to the survey applied to the Latin American rheumatologists, we conclude that, despite the recognized importance, in LA, not only economic, but also linguistic, cultural, social, and gender barriers can present challenges and barriers for the healthcare system and its policy for the treatment and health access in the context of ERA.

This gives us a picture of the overall setting in which the health system reforms are being carried out and how it changes RA care access. We should also add data concerning the resources for health systems, to have a better representation of the possibilities and constraints in realizing the health system reforms, specifically in terms of ERA care policy.
